# Efficacy and Safety of Axicabtagene Ciloleucel and Tisagenlecleucel Administration in Lymphoma Patients With Secondary CNS Involvement: A Systematic Review

**DOI:** 10.3389/fimmu.2021.693200

**Published:** 2021-07-05

**Authors:** XiaoQin Wu, XinYue Zhang, RenDe Xun, MengSi Liu, Zhen Sun, JianChao Huang

**Affiliations:** ^1^ Department of Neurosurgery, The First Affiliated Hospital, University of South China, Hengyang, China; ^2^ College of Integrated Chinese and Western Medicine, Affiliated Hospital of Traditional Chinese Medicine, Southwest Medical University, Luzhou, China; ^3^ Hengyang Medical College, University of South China, Hengyang, China

**Keywords:** CAR-T cell therapy, secondary CNS lymphoma, axicabtagene ciloleucel, tisagenlecleucel, safety, efficacy

## Abstract

**Background:**

The efficacy and safety of chimeric antigen receptor T (CAR-T) cell therapy in the treatment of non-Hodgkin’s lymphoma has already been demonstrated. However, patients with a history of/active secondary central nervous system (CNS) lymphoma were excluded from the licensing trials conducted on two widely used CAR-T cell products, Axicabtagene ciloleucel (Axi-cel) and Tisagenlecleucel (Tisa-cel). Hence, the objective of the present review was to assess whether secondary CNS lymphoma patients would derive a benefit from Axi-cel or Tisa-cel therapy, while maintaining controllable safety.

**Method:**

Two reviewers searched PubMed, Embase, Web of Science, and Cochrane library independently in order to identify all records associated with Axi-cel and Tisa-cel published prior to February 15, 2021. Studies that included secondary CNS lymphoma patients treated with Axi-cel and Tisa-cel and reported or could be inferred efficacy and safety endpoints of secondary CNS lymphoma patients were included. A tool designed specifically to evaluate the risk of bias in case series and reports and the ROBINS-I tool applied for cohort studies were used.

**Results:**

Ten studies involving forty-four patients were included. Of these, seven were case reports or series. The other three reports were cohort studies involving twenty-five patients. Current evidence indicates that secondary CNS lymphoma patients could achieve long-term remission following Axi-cel and Tisa-cel treatment. Compared with the non-CNS cohort, however, progression-free survival and overall survival tended to be shorter. This was possibly due to the relatively small size of the CNS cohort. The incidence and grades of adverse effects in secondary CNS lymphoma patients resembled those in the non-CNS cohort. No incidences of CAR-T cell-related deaths were reported. Nevertheless, the small sample size introduced a high risk of bias and prevented the identification of specific patients who could benefit more from CAR-T cell therapy.

**Conclusion:**

Secondary CNS lymphoma patients could seem to benefit from both Axi-cel and Tisa-cel treatment, with controllable risks. Thus, CAR-T cell therapy has potential as a candidate treatment for lymphoma patients with CNS involvement. Further prospective studies with larger samples and longer follow-up periods are warranted and recommended.

## Introduction

In contrast to primary central nervous system (CNS) lymphoma, secondary CNS lymphoma is caused by a lymphoma that originated elsewhere and metastasized to the CNS. The latter malignancy is usually associated with a dismal prognosis (1–3). Currently, high-dose methotrexate-based chemotherapy, targeted therapy, whole-brain radiation therapy, and consolidative autologous hematopoietic stem cell transplantation (HSCT) may enable lymphoma patients with CNS involvement to achieve long-term survival. However, patient relapse and drug resistance development lead to devastating outcomes by reducing patient survival to only a few months, in the absence of chimeric antigen receptor (CAR-T) cell therapy (4–11). CAR T-cells are genetically engineered autologous or allogeneic T cells that are used to target specific types of tumor cells. They have demonstrated remarkable efficacy and acceptable safety in the management of relapsed or refractory (r/r) B-cell malignancies both in clinical trials as well as in real-world applications (12–16).

The CAR-T cell products, Axicabtagene ciloleucel (Axi-cel) and Tisagenlecleucel (Tisa-cel), which are used to target CD19, were approved by the U.S. Food and Drug Administration in 2017, and currently account for a majority of CAR-T cell use in the United States (17, 18). In the Axi-cel and Tisa-cel licensing trials, 58 and 40% of all patients with r/r large B-cell lymphoma (LBCL), respectively, achieved complete response (CR), and significantly prolonged overall survival (OS) and progression-free survival (PFS) were observed. However, due to concerns that CNS disease may increase the incidence and severity of immune effector cell-associated neurotoxicity syndrome (ICANS), patients with a history of prior CNS lymphoma or active secondary CNS lymphoma at the time of CAR-T infusion were excluded from the Axi-cel and Tisa-cel licensing trials, respectively (16, 19).

Sixty-four percent of the patients in the ZUMA-1 trial conducted on Axi-cel, using CD28 as the co-stimulation domain, presented with any grade ICANS, with 28% exceeding grade 2. ICANS typically manifests as CNS dysfunctions, including language disturbance, impaired handwriting, disorientation, altered levels of consciousness, seizures, motor weakness, cerebral edema, and increased intracranial pressure, some of which are potentially fatal (20). The exact mechanism underlying ICANS pathology has not been elucidated. Previous studies have indicated that it may be associated with endothelial activation in the CNS, increased permeability of the blood–brain barrier, and upregulated levels of inflammatory factors (21–25). A recent study reported that targeting of CD19^+^ brain mural cells may contribute to neurotoxicity in CAR-T therapy (26). Presently, no agent is completely effective at mitigating ICANS. Clinicians often resort to glucocorticoid administration for the treatment of this condition. Nevertheless, these drugs may impair CAR-T cell efficacy and increase the risk of infection (23, 27, 28). Therefore, clinicians and decision makers exercise caution in the treatment of lymphoma patients with CNS involvement and often exclude them altogether as candidates for CAR-T therapy.

For r/r lymphoma patients with CNS involvement, however, CAR-T cell therapy may be their last and only potentially beneficial treatment option. For this reason, it is important to perform a risk-benefit analysis of Axi-cel and Tisa-cel therapy in these patients. Presently, research on CAR-T cell therapy for lymphoma patients with CNS involvement has been conducted at various institutions. The efficacy and the safety profiles and clinical characteristics of this treatment approach have not been comprehensively reported. In the present review, we extensively searched current studies of all types on Axi-cel and Tisa-cel, reviewed studies that included patients with CNS involvement, and summarized treatment efficacy and safety endpoints. The objectives of this review were to provide a comprehensive and systematic description and evaluation of CAR-T cell therapy for lymphoma patients with CNS involvement and to facilitate its future clinical administration.

## Methods

Our reporting items followed the Preferred Reporting Items for Systematic Reviews and Meta-Analyses (PRISMA 2020) guidelines (29). The protocol for this study was not registered.

### Search Strategies

On February 15, 2021, two reviewers independently searched PubMed, Embase, Web of Science, and Cochrane library for studies related to Axi-cel and Tisa-cel. We used the search terms “axicabtagene ciloleucel”, “axi-cel”, “kte c19 car”, “kte c19”, “ktec19”, “yescarta”, “tisagenlecleucel”, “kymriah”, and “CTL019” without filters or language restrictions. The reference lists in the included articles and relevant reviews, were also reviewed.

### Eligibility Criteria

Reports meeting several specific criteria were considered for inclusion in this review: (a) Some proportions, or the entirety, of a study population that presented with lymphoma with secondary CNS involvement, regardless of whether CNS disease had been cleared or was active at the time of CAR-T cell infusion; (b) All patients were treated with Axi-cel or Tisa-cel; (c) Studies that reported the number or grade of any adverse effects, including ICANS, cytokine release syndrome (CRS), or any efficacy endpoints including CR, overall response (OR), OS, or PFS in patients with CNS involvement; and (d) All clinical studies were eligible including case reports and cohorts, case-control, single-arm, prospective, and retrospective studies.

Reports meeting certain other criteria were excluded: (a) Studies without original data, including reviews, meta-analyses, clinical study protocols, comments or editorials; (b) Studies involving nonhuman research subjects and molecular, cellular, or animal experiments; (c) Studies in which subjects were included on the basis of specific efficacy or safety endpoints such as retrospective studies conducted on patients with CRS; (d) Studies that did not report or could not be inferred endpoints of interest for lymphoma patients with CNS involvement; (e) Reports including populations that overlapped those of other studies; and (f) Studies wherein patient disease was not an approved indication.

The institutions and study periods pertaining to patients included in each report meeting the foregoing eligibility criteria were reviewed to avoid overlapping of cases. When cases were found to overlap, the studies with relatively fewer patients were omitted. However, all reports that addressed overlapping patients, but reported different endpoints of interest, were included in the review.

### Selection and Data Extraction

Based on the inclusion and exclusion criteria, two reviewers independently screened all initially retrieved records. The reasons for exclusion were recorded and checked against the list of excluded literature.

For the eligible studies, two reviewers independently extracted data pertaining to the first author, publication year, product type, number of patients with CNS involvement, age, gender, disease type, CNS disease status, CAR-T cell dosage, bridging therapy, lymphodepletion regimen, prior treatment lines, follow-up time, response status, survival time, PFS, toxicity grading scale, and type, as well as the number and grade of adverse events. The last reported time for endpoints of interest was considered as the follow-up time. Certain included patients were a subgroup in cohort studies that did not specifically provide data on the baseline characteristics for patients with CNS involvement. Hence, the age range and prior lines of treatment in the total cohort were used as reference. Any discrepancy in data extraction was resolved by consensus *via* discussion.

### Study Bias Risk Assessment

Two reviewers independently assessed the methodological quality of the included studies. Conflicts that arose were resolved by discussion. In regard to case reports and series, a tool comprising eight items was applied to evaluate uncontrolled case series and case reports focusing on patient selection, ascertainment of exposure and outcome, causality, and reporting of data (30). Dose–response effects and challenge/rechallenge phenomenon in the causality domain were not applicable to the included studies. Thus, neither aspect was evaluated. Patient selection was deemed critical for reviewing validity. A study was considered to be at high risk of bias if it did not explicitly state that the cases that were presented constituted all cases at that institution over the time period concerned. Moreover, studies failing to meet more than two other criteria were also considered to be at high risk of bias, while the rest were classified as low risk of bias. The modified quality assessment tools are presented in **Table 1**. For cohort studies, the Risk Of Bias In Non-randomized Studies of Interventions (ROBINS-I) tool that includes seven domains termed as cofounding, selection, classification of interventions, deviations from intended interventions, missing data, and measurement of outcomes and reported results, was used. Risk of bias was rated as low, moderate, serious, or critical (31).

**Table 1:** The quality assessment tool for case reports and case series.

Domains	Leading explanatory questions
**Selection**	– Does the patient(s) represent(s) the whole experience of the investigator (center) or is the selection method unclear to the extent that other patients with similar presentation may not have been reported?
**Ascertainment**	– Was the exposure adequately ascertained? – Was the outcome adequately ascertained?
**Causality**	– Were other alternative causes that may explain the observation ruled out? – Was follow-up long enough for outcomes to occur?
**Reporting**	– Is the case(s) described with sufficient details to allow other investigators to replicate the research or to allow practitioners make inferences related to their own practice?

## Results

**Figure 1:**
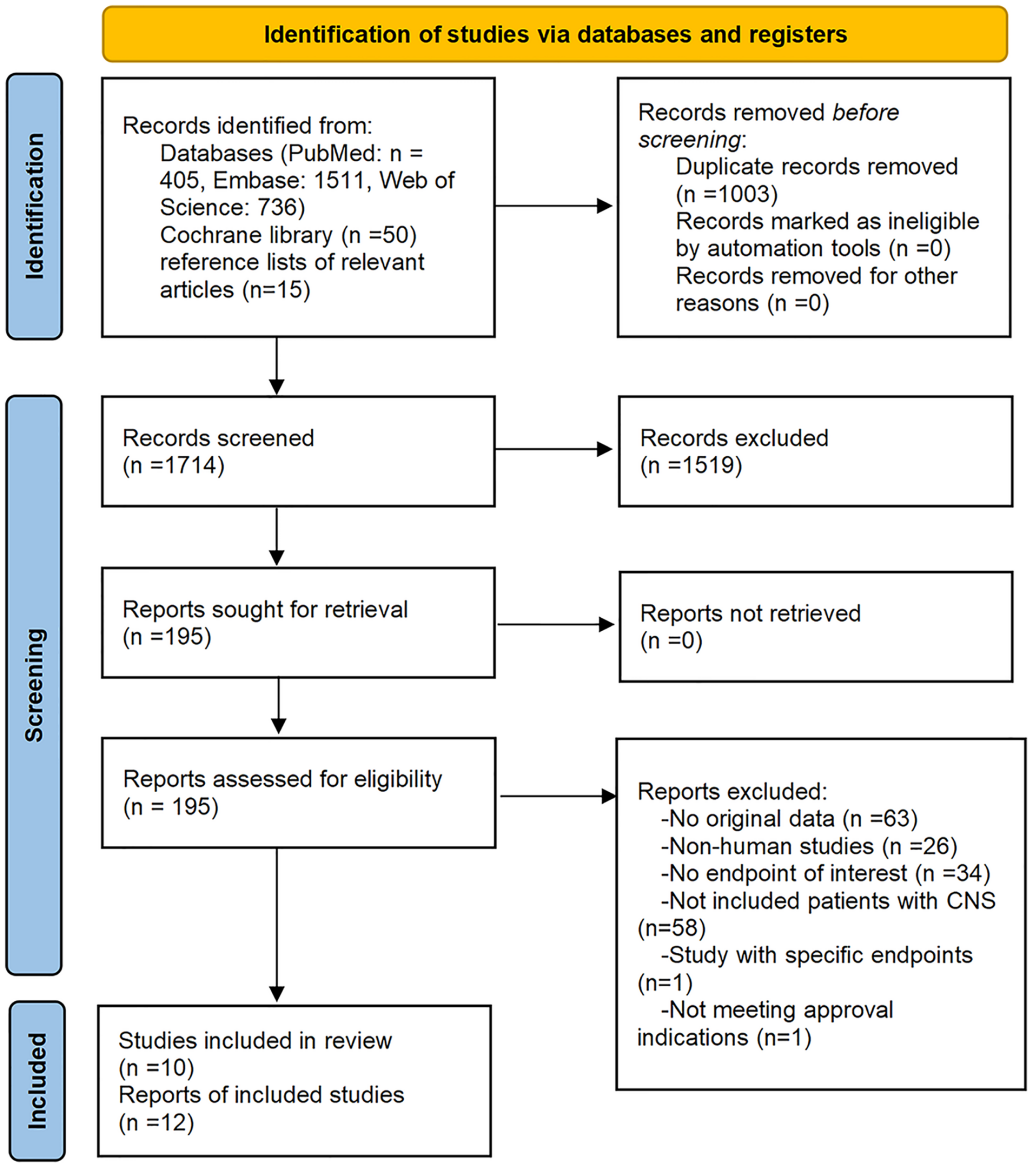
Flow diagram of the study select process.

Initially, our literature search yielded 2,717 records. Removal of duplicate literature and screening of titles and/or abstracts resulted in 2,522 records being excluded. Full texts of the remaining 195 records were reviewed. Ten studies met the eligibility criteria. Two of the ten studies were reported twice and stated somewhat different endpoints of interest (14, 32–42). Detailed selection flow and the reasons for exclusion are shown in **Figure 1**.

### Study Characteristics

After excluding overlapping patients, we identified 10 studies comprising 44 lymphoma patients with secondary CNS involvement. The patients in three studies belonged to a multicenter cohort study subgroup (14, 32, 35–37). All other studies were either case reports or series (33, 34, 38–42). Axi-cel was administered to thirty-five patients in eight studies (14, 32–40, 43), while Tisa-cel was admministed to nine patients in the remaining two studies (41, 42). Thirteen patients presented with diffuse large B-cell lymphoma (DLBCL), four with high-grade B-cell lymphoma (HGBCL), two with primary mediastinal B-cell lymphoma (PMBCL), and twenty-five with LBCL of no specified subtype. All patients, except one, were adults aged at least 18 years. The reported lymphodepleting chemotherapy regimen was consistent with those reported in the ZUMA-1 and JULIET trials. For Axi-cel, fludarabine (30 mg/m^2^) and cyclophosphamide (500 mg/m^2^) were administered daily for three days. For Tisa-cel, cyclophosphamide (250 mg/m^2^) and fludarabine (25 mg/m^2^) were administered daily for three days (16, 19). Three studies reported CAR-T cell doses ranging from 0.6–6.0 × 10^8^, or 2 × 10^6^/kg. All patients except one received at least two other forms of systemic treatment. Bridging therapy was comprised of systemic and radiation therapies. In four studies, duration of the longest follow-up was a period of more than six months (14, 32, 38, 39, 42). Three studies were conducted for less than six months (33, 40, 41). The duration periods of the remaining three studies could not be determined (34–37). At the time of Axi-cel infusion, CNS disease was active in 11 patients but resolved in 20 others. Eight patients presented with active CNS disease at the time of Tisa-cel infusion. The CNS status of the other patients was unclear. Three studies specified whether individual patients had concomitant systemic diseases (38, 39, 41). Detailed information pertaining to the study is shown in **Table 2**.

**Table 2:** The baseline characteristics of included studies.

Studies	Product	No.	Median age (range)-yr.	Male	Histological type (s)	Prior line of therapy	CAR-T cell dose	Bridging therapy	Lymphodepletion	CNS disease at time of CAR-T infusion	ICANS (scale)	CRS (scale)	Response status	Survival data	Follow-up
**Nastoupil** ^a^ (14)	Axi-cel	21*	(21–83) ^	NP	LBCL	(2–11) ^	NP	NP	NP	NP	Grade ≥3: 7 (43%)^†^ (CTCAEv4.03 or CARTOX)	Grade ≥3: 3 (17%)^†^ (CARTOX or Lee)	CR: 9(50%)^†^	PFS Rate at 12 Ms: 44%, OS Rate at 12 Ms: 56%^†^	≤12 Ms
**Bennani** ^a^ (32)	Axi-cel	17*	58 (25–83)	11	LBCL	4 (2–6)	NP	12/17 ST, 2/17 RT, 1/17 steroids only	NP	5: active, 10 resolved	Any grade: 13/15, grade ≥3: 5/15 (CTCAEv4 or CARTOX)	Any CRS: 14/15, grade 3 CRS: 2/15 (Lee criteria)	OR: 10/17	Median PFS: 3.0 (1.6-NE) (ITT analysis with 17 patients)	≤12 Ms
**Jain** (33)	Axi-cel	1	56	1	DLBCL	3	NP	Ibrutinib with ICE	Flu/Cy	NP	None (NP)	None (NP)	None	Dead on day 77	77 Ds
**Abbasi** (34)	Axi-cel	2	(55–77) ^	NP	DLBCL	(2–4) ^	NP	NP	Flu/Cy	NP	NP	NP	CR: 2	PFS: ≥5 Ms	≥5 Ms
**Holtzman** (35)	Axi-cel	2	(26–75) ^	NP	LBCL	NP	NP	NP	NP	Resolved	None (CTCAE 4.03)	NP	NP	NP	NP
**Strati** ^b^ (36)	Axi-cel	8	(18–85) ^	NP	LBCL	(2–15) ^	2 × 10^6^ cells/kg	NP	Flu/Cy	Resolved	Any grade: not evaluated grade≥3: 5 (CARTOX)	NP	NP	NP	NP
**Strati** ^b^ (37)	Axi-cel	8	(18–85) ^	NP	LBCL	(2–15) ^	NP	NP	NP	Resolved	NP	NP	NP	Median PFS: 2 Ms Median OS: 3 Ms	NP
**Ghafouri** (38)	Axi-cel	5	59 (28–76)	NP	2 HGBCL, 2 DLBCL, 1 PMBCL	2.4 (1–4)	NP	3 patients administered^※^	NP	Active	1 grade 3, 1 grade 4 (NP)	1 grade 1, 1 grade 2 (NP)	3 CR, 1 SD, 1 PD	Median PFS: 134 Ds Median OS: 155 Ds^#^	155 Ds (86–208)
**Novo** (39)	Axi-cel	1	62	1	DLBCL	3	NP	MTX and rituximab	NP	Active	NP^¶^	Grade 2 (Lee)	CR	PFS≥12 Ms	≥12 Ms
**Shah** (40)	Axi-cel	1	53	1	DLBCL	NP	NP	NP	NP	NP	Grade 4 (CARTOX)	NP	NP	NP	10 Ds
**Frigault** (41)	Tisa-cel	8	50 (17–79)	4	5 DLBDL, 2 HGBCL, 1 PMBCL	5 (3–6)	0.6–6.0 × 10^8^ cells	8 patients administered	Flu/Cy	Active	3 grade 1 ^§^ (Lee, ASTCT/Lee, ASTCT)	7 grade 1 (Lee, ASTCT/Lee, ASTCT)	3 CR, 1 PR, 4 PD	NP	≤6 Ms
**Hrosler** (42)	Tisa-cel	1	19	NP	DLBCL	2	0.9 × 10^8^ cells	NP	NP	NP	Grade 2 (NP)	Grade 1 (NP)	OR^‡^	PFS: 10 Ms	10 Ms

CNS, central nervous system; Axi-cel, Axicabtagene Ciloleucel; Tisa-cel, Tisagenlecleucel; LBCL, large B-cell lymphoma; DLBCL, diffuse large B cell lymphoma; PMBCL, primary mediastinal B-cell lymphoma; HGBCL, high-grade B cell lymphoma; CAR-T, chimeric antigen receptor T; NP, not provided; CRS, cytokine release syndrome; ICANS, immune effector cell-associated neurotoxicity syndrome; Flu/Cy, fludarabine/cyclophosphamide; CR, complete response; PR, partial response; OR, overall response; PD, progressive disease; SD, stable disease; OS, overall survival; PFS, progression-free survival; Ms, months; Ds, days; ST, systemic therapy; RT, radiation therapy; ICE, ifosfamide, carboplatin and etoposide; RDHAX, rituximab, dexamethasone, cytarabine, oxaliplatin; MTX, methotrexate; ITT, intention to treat; ASTCT, American Society for Transplantation and Cellular Therapy criteria; CARTOX, CAR-T-cell-therapy-associated TOXicity; CTCAE, Common Terminology Criteria for Adverse Events.

^†^The number of patients evaluated was not provided.

*Intention to treat patients.

^Range of the total cohort.

^※^The specific regimen was reported in the original paper.

^#^Of the responders.

^¶^No rating but related symptoms were described.

^‡^There was no report on whether the response was complete or partial.

^a,b^Reports from the same study.

^§^one patient was not evaluable due to disease progression.

In regard to case reports and series, five studies displayed high risks of bias. The main potential risk was caused by failure to specify whether the cases presented comprised all patients in the institution over a certain period, which may eventually lead to an overly optimistic assessment of the overall result (**Table 3**). Two of the three cohort studies presented with low bias risk, while the other presented with a moderate bias risk due to a lack of data on certain patients (**Table 4**).

**Table 3:** The quality assessment for included case reports or case series.

Study	Selection	Ascertainment		Causality		Reporting	Bias level
		Adequate exposure	Adequate outcome	Exclude other causes	Long enough follow-up		
**Jain**	no	yes	yes	yes	yes	yes	High risk
**Abbasi**	yes	yes	no	yes	no	no	High risk
**Ghafouri**	yes	yes	yes	yes	yes	yes	Low risk
**Novo**	no	yes	yes	yes	yes	yes	High risk
**Shah**	no	yes	yes	yes	no	yes	High risk
**Frigault**	yes	yes	yes	yes	no	yes	Low risk
**Hrosler**	no	yes	yes	no	yes	yes	High risk

**Table 4:** The quality assessment for included cohort studies.

Study	Confounding	Selection	Classification of intervention	Deviations from interventions	Missing data	Measurement of outcomes	Reported results	Bias level
**Nastoupil/Bennani**	Low risk	Low risk	Low risk	Low risk	Moderate risk*	Low risk	Low risk	**Moderate risk**
**Strati**	Low risk	Low risk	Low risk	Low risk	Low risk	Low risk	Low risk	**Low risk**
**Holtzman**	Low risk	Low risk	Low risk	Low risk	Low risk	Low risk	Low risk	**Low risk**

*The number of patients in two reports from this study is not same.

### Immune Effector Cell-Associated Neurotoxicity Syndrome

Fifteen of the twenty-three patients in the Axi-cel studies possessed any grade of ICANS (32, 33, 35, 38). Twelve of the thirty-one patients developed grade ≥3 ICANS (32, 33, 35, 36, 38). In a real-world multicenter cohort study conducted by Bennani et al., the grade ≥3 ICANS rates were comparable for fifteen LBCL patients (of whom ten presented with resolved CNS involvement and five with active CNS disease) and other patients with non-CNS involvement (33 and 31%, respectively). The incidences of any grade of ICANS were also comparable. No cerebral edema or seizures were observed. The use of glucocorticoid and tocilizumab in both the CNS and non-CNS cohorts were similar. However, compared to patients with a prior CNS history, those with active CNS disease at the time of CAR-T cell infusion tended to present with higher rates of grade ≥3 ICANS rate (60 *vs*. 20%, respectively) possibly as a result of the bias introduced by the small sample size (32). A patient with a history of CNS involvement reported in the study conducted by Jain et al., as well as two patients with CNS disease clearance before Axi-cel infusion reported in the study conducted by Holtzman et al., did not present with ICANS (33, 35). The study conducted by Strati et al., included eight patients with no clinically active CNS disease at the time of Axi-cel infusion. However, five of the eight experienced grade ≥3 ICANS and seven were administered glucocorticoids. Relative to patients with bone marrow biopsy and head & neck involvement, patients with CNS involvement tended to display a higher rate of severe ICANS (22.7% *vs*. 45.0% *vs*. 62.5%, respectively) (36, 37). In the study conducted by Ghafouri et al., which focused on five patients with active secondary CNS involvement, two experienced grades 3 and 4 ICANS, respectively. These patients were administered glucocorticoids combined with dual anti-epileptics, upon which their ICANS symptoms resolved with no long-term neurotoxic sequelae (38). Novo et al., reported one patient with systemic disease and a lesion of the left periventricular medial temporal lobe. After being administered Axi-cel, the patient presented with mild neurological symptoms that spontaneously improved without intervention; there was no cerebral edema. On day 11 after infusion, a relapse leading to right-side weakness and dysphagia prompted the use of glucocorticoids. The symptoms improved within 48 hours and completely resolved by day 16 (39). Shah et al., reported one patient with left parietal leptomeningeal spread who experienced grade 4 ICANS and acute infarctions in the anterior and posterior cerebral artery territories. After receiving osmotherapy for cerebral edema, the patient regained normal cognitive function on day 10 but incomplete right homonymous hemianopsia persisted (CARTOX score = 10/10) (40).

Of the nine patients in the Tisa-cel studies, four presented with grade <3 ICANS. No severe ICANS occurred (41, 42). In the study conducted by Frigault et al., three out of eight patients who received heavy pretreatment experienced grade 1 ICANS including headache, neuropathy, or tremors. Administering tocilizumab or glucocorticoids was not required for any patient. However, one patient succumbed to progressive disease (PD). The other two cases resolved spontaneously. Another patient in the study was not assessed for neurotoxicity because of PD. Notably, all patients were administered prophylactic anticonvulsants (41). Hrosler et al., reported that one patient with post-transplant lymphoproliferative disease of the CNS presented with grade 1 ICANS following Tisa-cel infusion, and the condition of the patient resolved spontaneously without intervention (42).

### Cytokine Release Syndrome

Four Axi-cel studies evaluated the incidence of CRS in 22 patients. Seventeen patients presented with any grade of CRS and two experienced grade ≥3 CRS (32, 33, 38, 39). Bennani et al., reported that the incidences of any grade of CRS (93% *vs*. 91% for the CNS and non-CNS cohort, respectively) and grade ≥3 CRS (13% *vs*. 6% for the CNS and non-CNS cohort, respectively) were not affected by CNS involvement (32). None of the patients in the studies conducted by Jian et al., Ghafouri et al., or Novo et al., presented with CRS worse than grade 2. All patients with CRS responded to supportive treatment or tocilizumab, while some cases resolved spontaneously without intervention (33, 38, 39).

Two Tisa-cel studies comprising nine patients assessed the incidence and severity of CRS. Eight patients presented with grade 1 CRS and no specific intervention was required (41, 42).

### Response to CAR-T Cells

In the five Axi-cel studies evaluating the response status, 18/26 patients achieved OR. These included two patients who were included in the intention-to-treat analysis but did not receive Axi-cel infusion. Moreover, 15/27 patients achieved CR (14, 32–34, 38, 39). In the intention-to-treat analysis, Bennani et al., found no significant difference between CNS and non-CNS cohorts, in terms of the best OR rate (59% *vs* 75%, respectively; p = 0.15), the ongoing OR rate at three months (31% *vs*. 54%, respectively; p = 0.12) or the ongoing rate at six months (31% *vs*. 41%, respectively; p = 0.60) (32). CNS and non-CNS cohorts were also statistically similar in terms of their best CR rates at 12 months (50 and 65%, respectively; p = 0.21) (14). In the five patients with active CNS disease, one showed a partial response while two achieved CR. Among those presenting with resolved CNS disease, two PD patients both occurred systemically. In the study conducted by Abbasi et al., two patients with DLBCL of prior CNS involvement achieved CR for more than five months at data cutoff (34). Studies conducted by Ghafouri et al., and Novo et al., evaluated the responses of patients with CNS involvement. One patient was a refractory case in which a systemic or CNS response could not be detected, and another patient only achieved stability in the CNS disease. All others presented with CR of CNS (38, 39). Jain et al., reported that a patient who had undergone PD prior to lisocabtagene maraleucel treatment and presented with no response after Axi-cel infusion (33).

Five of nine patients in the two Tisa-cel studies achieved OR, while three of the eight patients achieved CR (41, 42). In the study conducted by Frigault et al., three patients achieved CR, one presented with PR, and the remaining four presented with PD (41). One case reported by Hrosler et al., achieved PR following pembrolizumab treatment. Subsequent Axi-cel infusion deepened and consolidated this response (42).

### Progression-Free Survival and Overall Survival

Seven Axi-cel studies reported survival data for lymphoma patients with CNS involvement (14, 32–34, 37–39). The study conducted by Nastoupil et al., showed that the PFS and OS rates for CNS and non-CNS cohorts at 12 months were 44% *vs*. 47% (p = 0.23) and 56% *vs*. 69% (p = 0.21), respectively, and these differences were not significant (14). The median PFS of CNS and non-CNS cohorts was 3.0 months (95%CI: 1.6–not estimable) and 9.5 months (95%CI: 6.2–18.8), respectively, and the hazard ratio was 1.65 (95% CI: 0.81–3.37) (32);. The study conducted by Strati et al., reported that the PFS and OS between prior and non-prior CNS patients were two months *vs*. eight months (p = 0.95) and three months *vs*. not reached (p = 0.35), respectively. Hence, the prior CNS patients tended to have shorter survival but remained comparable (37). In the study conducted by Ghafouri et al., the median PFS and OS in the responders were 134 and 155 days, respectively. Two of the four responders without concurrent systemic disease experienced a recurrence of CNS around day 140 and subsequently succumbed to PD around day 200. Another responder with concurrent systemic disease experienced a systemic only relapse on day 80, followed by death due to cardiopulmonary failure. The remaining responder with concurrent systemic disease maintained PFS for over six months followed by an allogeneic hematopoietic stem cell transplant. The patient was in sustained remission at the time of submission (38). All cases reported by Abbasi et al., and Novo et al., were maintaining PFS at data cutoff. One patient was followed-up for approximately 12 months, while the other two were followed-up for over five months. One case reported by Jian et al., failed to respond and succumbed to sepsis on day 77 in a rapid PD setting (33, 34, 39).

In the study conducted by Frigault et al., two patients without systemic disease died within 30 days of receiving Tisa-cel infusion. The autopsy showed that the cause of death was PD rather than CAR-T-driven toxicity. Two patients without systemic disease achieved PR and CR, respectively, and sustained responses for 90 days. One patient without systemic disease achieved PR on day 28 and CR on day 180 without concomitant maintenance. One patient with systemic disease achieved CR, but experienced a systemic relapse at day 90, which was subsequently resolved *via* radiotherapy. Overall, all patients responding to CAR-T cells were maintaining a response even until the very point of data cutoff. Nevertheless, the follow-up duration was short. Moreover, one patient with PD had concomitant systemic disease at the time of CAR-T cell infusion, while another with PD did not (41). A patient reported by Hrosler et al., remained in good condition for 10 months after Tisa-cel infusion (42).

## Discussion

To the best of our knowledge, this systematic review is the first to evaluate the efficacy and safety of CAR-T cell products in lymphoma patients with secondary CNS involvement. Compared with those examined in pivotal trials, patients with secondary CNS lymphoma treated with CAR-T cell products presented with similar rates for any grade of ICANS (Axi-cel in CNS cohort: 56% (15/23) *vs*. 64% in ZUMA-1; Tisa-cel in the CNS cohort: 50% (4/8) *vs*. 21% in JULIET), grade ≥3 ICANS [Axi-cel in the CNS cohort: 39% (12/31) *vs*. 28% in ZUMA-1; Tisa-cel in the CNS cohort: 0% (0/8) *vs*. 12% in JULIET], any grade of CRS [Axi-cel in the CNS cohort: 77% (17/22) vs. 93% in ZUMA-1; Tisa-cel in the CNS cohort: 89% (8/9) vs. 58% in JULIET (Penn scale)], and grade ≥3 CRS [Axi-cel in the CNS cohort: 9% (2/22) *vs*. 13% in ZUMA-1; Tisa-cel in the CNS cohort: 0% (0/9) *vs*. 22% in JULIET (Penn scale)] (16, 19). No CAR-T cell-related deaths were observed. In patients with secondary CNS lymphoma who were administered CAR-T cell products, the OR and CR rates were also similar to those for patients examined in the pivotal trials. The median PFS and OS of the CNS cohort showed a shorter trend compared with those of the non-CNS cohort. Nevertheless, the values were still comparable, which may be attributed to the small size of the CNS cohort (32, 37). Overall, the current studies indicated that lymphoma patients with CNS involvement could potentially achieve long-term remission in response to CAR-T cell products, and no conclusive evidence indicating that PFS or OS was worse for this group than that for patients without CNS involvement has been found.

Glucocorticoid and tocilizumab treatment in the CNS cohort resembled that seen in the non-CNS cohort, indicating that both groups had similar potential of developing severe CRS or ICANS. Limited data suggested that CAR-T cell therapy could achieve a sustained response and acceptable safety in DLBCL, PMBCL, and HGBCL patients with CNS involvement. None of the studies included in the review had analyzed the effect of patient age or gender on endpoints. However, in studies reporting baseline demographics for each individual, improved responses could be observed across genders, in youth, and in patients over 60 years of age (38, 39, 41, 42). Despite relapsed or refractory disease after multiple lines of systemic therapy, a large proportion of patients responded to CAR-T-cells and maintained long-term remission (38, 39, 41, 42). Certain factors, including lactate dehydrogenase, the International Prognostic Index, the Eastern Cooperative Group performance status, tumor size, and double or triple hits, may be associated with the efficacy and toxicity of CAR-T cell therapy. But none of these factors were compared among cohorts or reported for individual patients with CNS disease. Furthermore, the reports were heterogeneous in terms of the study design. For example, in the study conducted by Frigault et al., all patients with Tisa-cel infusion were administered prophylactic anticonvulsants (41). In the studies conducted by Ghafouri et al., and Frigault et al., patients were administered CNS-directed therapy until lymphodepletion. Nevertheless, they did not exhibit significantly better responses compared to those reported in other studies (38, 41). As data were limited, it was impossible to assess the impact of the CAR-T cell dosage, bridging therapy, or post-CAR-T cell infusion concomitant maintenance on efficacy or safety.

The use of different toxicity scales among studies may have contributed to the differences observed in their toxicity profiles. In the Axi-cel study, either the CTCAEv4.03 or the CARTOX scale was used to evaluate ICANS. However, studies using CARTOX may overestimate severe neurotoxicity by upgrading factors such as convulsive seizure and focal motor weakness to grade 4 (36). Furthermore, Pennisi et al., found that CARTOX application may miss mild ICANS. These may partly explain the fact that compared with the ZUMA-1 trial using CTCAEv4.03, any grade of ICANS grade in patients with CNS involvement was slightly lowered while the incidence of severe ICANS was slightly increased (44). However, no symptoms or indicators of toxicity were provided following CAR-T cell infusion. Hence, it was difficult to infer the definitive impact of various toxicity scales on results. The use of different scales may confound pooled results and weaken comparisons among studies. Therefore, it may be necessary to implement a unified grading system in future studies.

Only three studies including patients with active CNS involvement reported the sites of CNS disease and specified which patient presented with concomitant systemic diseases at the time of CAR-T cell infusion. Responses to two commercial CAR-T cells were observed in patients who were parenchymal and/or leptomeningeal or simply CSF-positive. However, these markedly few cases and inadequate follow-up is impossible to determine which patients can get more favorable results from the treatment. Prior studies have confirmed that CAR-T cells are able to cross the blood-brain barrier (45, 46). In the reviewed studies, Ghafouri et al., and Frigault et al., found that both Axi-cel and Tisa-cel were sufficiently trafficked to the CNS and underwent expansion in patients with isolated CNS disease, indicating that active systemic disease was not a necessary condition for responses (38, 41). Toxicity was manageable and there were no significant differences among above patient subtypes. Notably, in Axi-cel treatment, patients with active CNS disease appeared to present with excess severe ICANS. Two of the five patients in the study conducted by Ghafouri et al., and three of the five in the study conducted by Bennani et al., presented with grade ≥3 ICANS. The CARTOX scale was used in both studies (32, 38). This phenomenon was not observed for CRS or in Tisa-cel treatment. Limited data that was available showed that the efficacy and the safety profiles for CAR-T cell therapy in patients with resolved CNS disease were similar to the overall values. Relapse and treatment failure data were also limited. In the studies conducted by Ghafouri et al., and Frigault et al., two out of the patients with concomitant systemic disease presented with isolated systemic relapses following responses. Two out of the patients without concomitant disease relapsed, both from the CNS (38, 41). The study conducted by Bennani et al., reported that two out of the patients with resolved CNS disease had presented with systemic PD (32).

T cell exhaustion, activation-induced cell death, and antigen loss may cause resistance to CAR-T cell therapy (47, 48). In addition, whether tumor cells in the CNS interfere with CAR-T cell infiltration or function remains unknown. Given in the case series reported by Ghafouri et al., secondary CNS lymphoma patients presented with short remission periods, while the study reported by Frigault et al. had a short follow-up period (38, 41). Therefore, incorporation of consolidated strategies may confer benefits to patients. Ibrutinib and lenalidomide are logical considerations for consolidative therapy as they were found to be effective against primary CNS lymphoma (49–51). It is unknown whether allogeneic HSCT is suitable for patients with non-Hodgkin’s lymphoma who have been administered CAR-T. Furthermore, there are also concerns that CNS immune privilege may impair the graft-versus-tumor effect. Sterling et al., found that the median OS and PFS of 21 secondary CNS lymphoma patients who did not receive CAR-T cells were greater than three years, and that these patients tolerated allogeneic HSCT treatment (52). In the study conducted by Ghafouri et al., the patient presenting with a sustained response for over six months was also administered allogeneic HSCT following Axi-cel infusion (38). However, current evidence does not encourage consolidation therapy, such as autologous or allogenic HSCT for patients who respond to CAR-T cell therapy, unless subsequent research confirms that secondary CNS involvement is a risk factor for disease progression or recurrence (53, 54).

Limited sample size was a main limitation of this study. The use of small sample sizes leads to more conservative estimates, in comparison to patients with non-CNS involvement in cohort studies. When sample sizes are large, differences in efficacy and toxicity may show significance. Next, the studies included in this review consisted mainly of case reports and series with low levels of evidence. Four studies did not explicitly describe whether the cases they presented comprised all cases in their institution over a certain time period. Hence, it is possible that certain researchers may have preferentially reported patients who were responding to treatments and exhibiting relatively better prognoses. However, each of the four studies reported only one case with secondary CNS lymphoma. If these were to be excluded, the conclusions would remain unaffected. Thirdly, certain studies did not focus on secondary CNS lymphoma patients. Thus, these studies failed to comprehensively describe baseline characteristics, study designs, or endpoints of interest. Heterogeneity in these aspects, especially the use of different toxicity grading systems among studies may confound pooled results. Finally, no long-term outcome with a longer than one year follow-up was reported. In the main study conducted on Tisa-cel, the longest follow-up period did not exceed six months. Studies on the administering of CAR-T cell products as a secondary CNS lymphoma therapeutic agent may advance our knowledge in this area. Nevertheless, it will be necessary to proceed with caution when formulating therapeutic decisions based on these findings.

## Conclusion

The present review revealed that administering the CAR-T cell products Axi-cel and Tisa-cel for the treatment of lymphoma patients with secondary CNS involvement seems to have promising efficacy and manageable adverse reactions. However, the current data were derived mainly from case series and reports pertaining to studies that used small sample sizes. Hence, we are unable to draw conclusions regarding which patient subsets may derive benefits more from this therapeutic approach. Prospective studies with large sample sizes and long follow-up periods are required to further explore the effects of factors predicting outcomes, CNS and systemic diseases statuses, and sites of CNS disease and for the formulation of strategies to counter relapses and treatment failures.

## Data Availability Statement

The original contributions presented in the study are included in the article/supplementary material. Further inquiries can be directed to the corresponding author.

## Author Contributions

JH and ZS contributed to the conception and design of the study. XW, RX searched the database. ML and RX extracted data. XZ, XW, and ZS wrote the first draft of the manuscript. All authors contributed to the article and approved the submitted version.

## Conflict of Interest

The authors declare that the research was conducted in the absence of any commercial or financial relationships that could be construed as a potential conflict of interest.
